# Rapid and Facile Fabrication of Polyiodide Solid-State Dye-Sensitized Solar Cells Using Ambient Air Drying

**DOI:** 10.1021/acsami.2c14299

**Published:** 2022-09-16

**Authors:** Matthew Sutton, Bingyu Lei, Hannes Michaels, Marina Freitag, Neil Robertson

**Affiliations:** † School of Chemistry, The University of Edinburgh, David Brewster Road, Edinburgh EH9 3FJ, U.K.; ‡ Department of Chemistry, Ångström Laboratory, Uppsala University, P.O. Box 523, SE-75120 Uppsala, Sweden; § School of Natural and Environmental Science, Bedson Building, Newcastle University, Newcastle upon Tyne NE1 7RU, U.K.

**Keywords:** solar cells, heterojunction, solid-state, polyiodide, stability

## Abstract

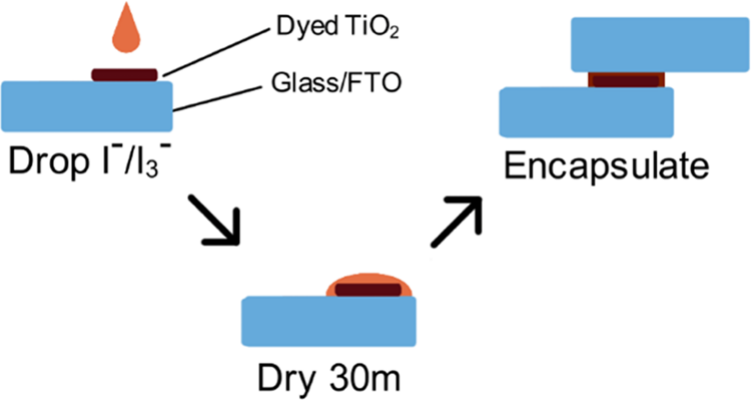

Dye-sensitized solar cells are promising candidates for low-cost indoor power generation applications. However, they currently suffer from complex fabrication and stability issues arising from the liquid electrolyte. Consequently, the so-called zombie cell was developed, in which the liquid electrolyte is dried out to yield a solid through a pinhole after cell assembly. We report a method for faster, simpler, and potentially more reliable production of zombie cells through direct and rapid drying of the electrolyte on the working electrode prior to cell assembly, using an iodide–triiodide redox couple electrolyte as a basis. These “rapid-zombie” cells were fabricated with power conversion efficiencies reaching 5.0%, which was larger than the 4.5% achieved for equivalent “slow” zombie cells. On a large-area cell of 15.68 cm^2^, over 2% efficiency was achieved at 0.2 suns. After 12 months of dark storage, the “rapid-zombie” cells were remarkably stable and actually showed a moderate increase in average efficiencies.

## Introduction

Dye-sensitized solar cells [DSSC] have the potential for low-cost and indoor power generation due to their earth-abundant materials and absorption profiles that are in the visible region.^1−7^ In this context, DSSCs have managed to surpass 30% power conversion efficiency [PCE] under visible light.^8^ However, they require further improvement in terms of long-term stability, ease of manufacture, and PCE.^9,10^

Most DSSCs use a liquid electrolyte, which generally gives higher PCE than solid-state and gel hole transport materials due to higher charge mobility,^9−11^ though they can suffer from leakage/evaporation, which can drastically lower cell performance over time.^2,3,7,10,12^ Solid hole transport materials and gel electrolytes that have been used often have poor interfacial contact and low conductivity, leading to a lower PCE.^2,3,11^ For example, cells fabricated by Leandri et al. using a solution iodide/triiodide redox electrolyte achieved 6.8% PCE, while their analogue cells using the solid-state hole transport material Spiro-OMeTAD achieved only 4.8%.^13^ Many solid-state DSSCs also use evaporated gold electrodes, which are costly and yield poor stability.^7,14,15^

Previous work by Freitag et al. in 2015 showed that “zombie cells” could be a potential solution.^9^ The zombie cell is a type of DSSC that continues to work after the electrolyte is dried out. The cell was assembled as a regular liquid cell, but after the Cu-complex electrolyte was injected, it was allowed to dry slowly through a small hole in the counter electrode. The slow, controlled drying allowed the formation of a solid hole transport material [HTM]. These cells had very good long-term stability due to avoiding evaporation/leakage of the predried electrolyte.^5,16^ A drawback of the zombie fabrication method is that, to date, the electrolyte has been dried slowly through a small hole over the course of a few days, impractical for any scale-up or manufacture.^9,10^ The issue with increasing the rate of drying is that with metal-complex HTMs (which to date have achieved the highest recorded PCE of 14% for DSSCs),^17^ this often leads to uncontrolled and undesired crystallization rather than the desired amorphous states. No alternative currently exists for quicker cell fabrication.

In this work, to speed up the drying process, we opted instead to use the iodide/triiodide (I^–^/I_3_^–^) redox couple, which was recently used to fabricate zombie cells by Tanaka et al. in 2020.^10^ Although it has not reached the same PCE as metal-complex HTMs due to *V*_OC_ loss,^16^ I^–^/I_3_^–^ is favored for use in DSSCs due to its simplicity and slow electron recombination kinetics.^3,10,12,18−20^ Also, we have previously shown that the zombie cells fabricated using the I^–^/I_3_^–^ redox couple have good long-term stability compared to equivalent liquid DSSCs.^10^

**Figure 1:**
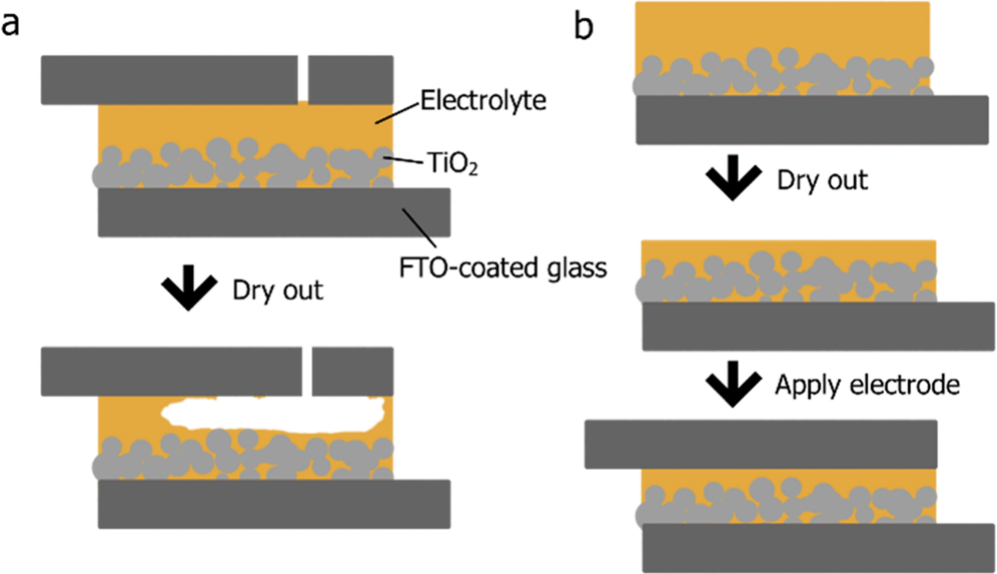
(a) Previously established drying process of “slow-zombie” (SZ) cell, with an exaggerated example of how the volume change during drying can create spaces in the electrolyte. (b) Drying process of an RZ cell, whereby the volume change happens before sealing, thus contact is maintained.

We report here that due to the amorphous nature of polyiodides, we were able to hasten the drying process by applying the electrolyte to the working electrode directly before cell assembly and allowing it to dry in ambient air for a short period of time. The counter electrode was added after drying was complete. These cells were labeled as “rapid-zombie” (RZ) cells. This method substantially increases the ease of fabrication, as there is no longer any need to drill an injection hole into the counter electrode, inject the electrolyte, or dry over several days (Figure 1). Due to the ease and robustness of the application procedure, other processing methods such as spray-coating could also be viable for these cells. This method has enabled extremely facile production of DSSCs, and with further development, rapid-zombie cells have the potential to become an easily manufactured, stable, low-cost form of indoor power generation for devices such as phones or as a replacement for disposable primary batteries.

## Experimental Section

Three types of cells were fabricated: rapid-zombie (RZ), slow-zombie (SZ), and liquid (Liq). Liq and SZ cells were made as described in the work of Tanaka et al.*,*^10^ but using the commercially available LEG4 dye in place of D149. RZ cells were fabricated by first dyeing the working electrode using the dye LEG4, then applying the electrolyte directly to the electrode and allowing it to dry in ambient air for 30 min. This drying time was not systematically tested, but 30 min was found to be suitably long enough to allow the solution to fully dry out. The electrolyte used in the Liq cells was as follows: I_2_ (0.05 M), LiI (0.1 M), 1,2-dimethyl-3-propylimidazolium iodide [DMPII] (0.6 M), anhydrous acetonitrile [ACN] (4.63 mL), and 4-*tert*-butylpyridine [TBP] (0.5 M, 0.37 mL).^10^ The SZ and RZ cell electrolytes were based on this formula, with both having double the I_2_ concentration (0.1 M I_2_) of the Liq formula and no LiI. These alterations were found to be favorable for the formation of a consistent film when dried on glass, where standard solutions with LiI or lower I_2_ concentrations formed patchy films, indicating poor coverage of the surface. Large-scale cells were fabricated with a doctor-bladed active area of 3.2 cm × 4.9 cm. These cells were fabricated using the RZ method and were dried for 1 h instead of 30 min before adding the counter electrode.

## Results and Discussion

**Figure 2:**
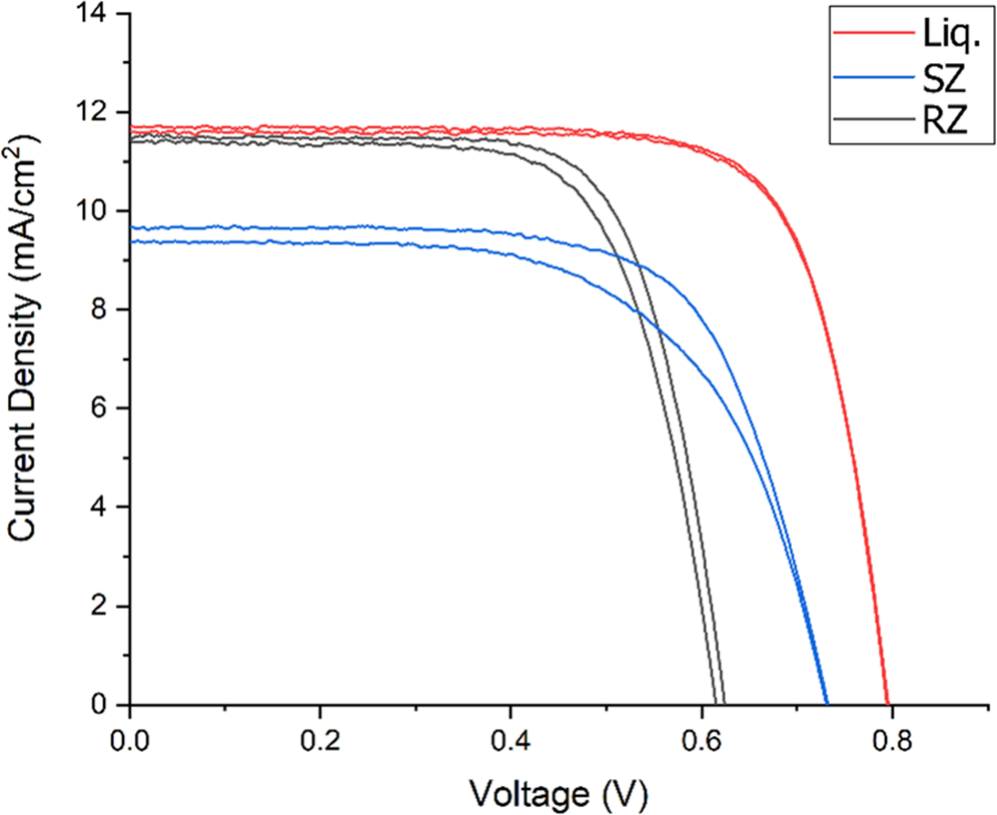
*J*–*V* curves for champion Liq, SZ, and RZ cells, recorded at 100 mW cm^–2^ using a 3 mm diameter (0.0707 cm^2^) circular mask between 0 and 0.8 V.

Figure 2 shows the *J*–*V* curves of the champion small-area RZ, SZ, and Liq cells, recorded at AM1.5 100 mW cm^–2^. The performance metrics for each cell type are shown in Table 1. *J*_SC_ was verified by IPCE (Figure S6). The champion Liq cell had a PCE of 7.0%. Leandri et al. achieved a PCE of 6.8% with a very similar LEG4 + iodide/triiodide cell structure in 2016,^13^ with which our results are consistent.

**Table 1:** Performance Metrics of Champion Liquid (Liq), Slow-Zombie (SZ), and Rapid-Zombie (RZ) Cells^a^

cell type	*J*_SC_ (mA cm^–2^)	*V*_OC_ (V)	fill factor (%)	PCE (%)
Liq	11.7	0.794	0.75	7.0
SZ	9.52	0.730	0.65	4.5
RZ	11.5	0.618	0.71	5.0

a Average values can be found in the Supporting Information (Table S1).

**Figure 3:**
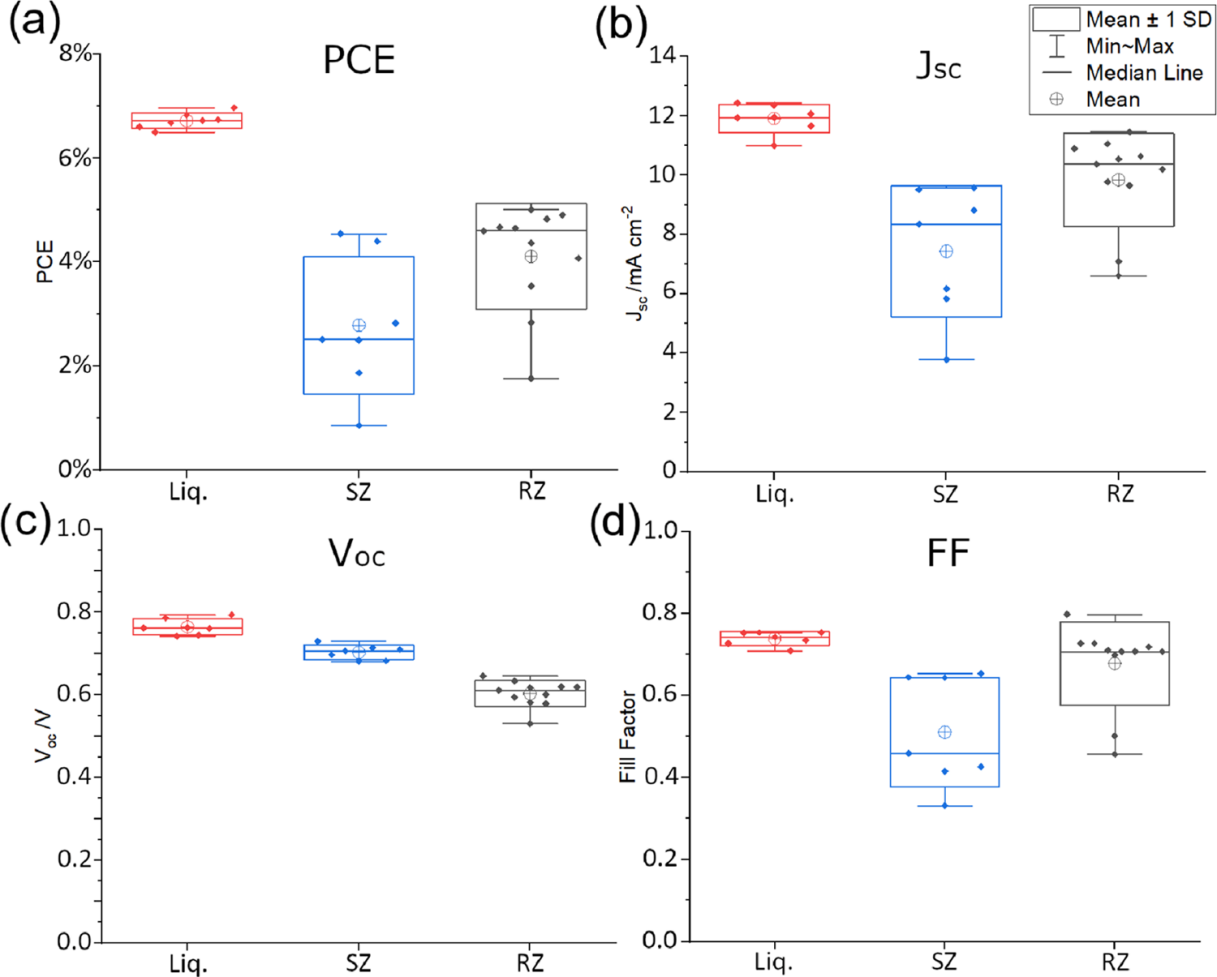
Box plots displaying the PCE (a), *J*_SC_ (b), *V*_OC_ (c), and fill factor (d) of the cells fabricated. Liq cells are displayed in red (left), SZ cells in blue (center), and RZ cells in black (right). Individual points represent a single cell. A few cells were omitted as they had values >3 standard deviations (calculated without outlying value) from the mean, attributed to human error. These comprised 1 Liq., 1 SZ, and 4 RZ cells of the 8, 8, and 16 fabricated, respectively.

A set of box plots (Figure 3) shows the performance of RZ cells in comparison to SZ cells and Liq cells and matches the trends seen for the champion cells. Overall, the performance of the RZ cells surpassed SZ cells, with the average PCE of RZ cells (4.1 ± 0.97%) being higher than the SZ cells (2.8 ± 1.2%). The larger errors for the RZ and SZ cells, compared to the more established liquid cells, may reduce over time as the novel fabrication method is further refined.

**Figure 4:**
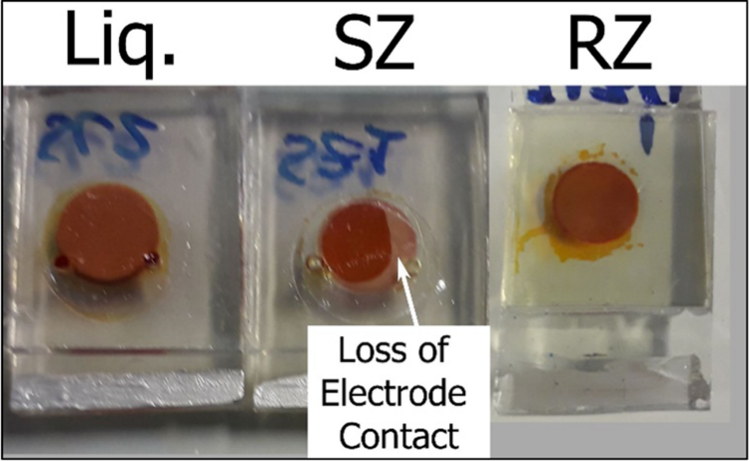
Image of the counter-electrode side of a Liq cell (left) and an SZ cell (center). The SZ cell was observed to have incomplete counter-electrode contact, as indicated by the lighter section of the active area, and the zone surrounding the active area was also evacuated of electrolyte, unlike the Liq or RZ cells.

The values of *J*_SC_ were of great interest, as the solidification of an HTM in a porous photoanode can lead to a lowering of the photocurrent since both pore-filling and dye accessibility play key roles in maintaining good *J*_SC_. This suggests that the faster drying method facilitated better pore-filling in the RZ cells than in the SZ cells, likely due to less occurrence of the phenomenon illustrated in Figure 1 for SZ cells, whereby the liquid electrolyte injected will dry out and subsequently reduce in volume. Since the cell is already sealed, this will inevitably lead to some areas without electrolytes contacting the electrodes. Therefore, SZ cells experienced an evacuation of electrolyte in the area surrounding the active area, and often it was observed that electrolyte was not contacting parts of the counter electrode. An example of the visual loss of interfacial contact can be seen in Figure 4. Better interfacial contact within the RZ cells, however, may also contribute to the ca. 100 mV lower *V*_OC_ over SZ cells through increased recombination, although the overall PCE remains higher. The lower *V*_OC_ of both the SZ and RZ cells compared with Liq cells may partially be attributed to the doubled [I_2_] in the zombie recipe. These *V*_OC_ losses will need to be mitigated for future device optimization.

Visible hysteresis was observed in both RZ and SZ cell types. Sarker et al. determined that hysteresis at short circuit (as seen for the SZ cells) indicates chemical capacitance at the fluorine tin oxide (FTO)/electrolyte interface, while hysteresis at open circuit (as seen for RZ cells) indicates chemical capacitance at the Pt/electrolyte interface.^21^ Hysteresis at the maximum power point indicates deficiencies in carrier abstraction from the TiO_2_/dye/HTM interface, which is seen most strongly for the SZ.

The long-term stability of each type of cell was tested over a period of 12 months in dark storage (ISOS-D-1).^22^ The results are shown in Table 2. RZ cells performed very well, actually seeing an average increase in performance of 12.2% over the year from 4.1 ± 0.97 to 4.6 ± 0.42%. This increase was attributed to the electrolyte being given time to slowly penetrate the mesoporous titania and form better interfacial contact. The SZ cells, on the other hand, saw an average change of 0%, with some cells improving and some decreasing in performance, going from 2.8 ± 1.22 to 2.8 ± 1.31% over the year. Predictably, the liquid cells all saw a sharp and consistent decrease, with cells going from 6.7 ± 0.14 to 1.4 ± 0.97%. This distinctly showed that the RZ cells were far more stable than their liquid counterparts and were overall more stable than their SZ equivalents, which used the same electrolyte formulation.

**Table 2:** Average PCE and Standard Deviation of Each Cell Type after 12 Months of Dark Storage^a^

cell type	original	after 12 months	% change
Liq	6.7 ± 0.14%	1.4 ± 0.97%	–79.6%
SZ	2.8 ± 1.22%	2.8 ± 1.31%	0%
RZ	4.1 ± 0.97%	4.6 ± 0.42%	+12.2%

a Details on individual performance metrics after 12 months of storage is available in Table S2.

Interestingly, some RZ cells which were originally omitted from the results (>3 standard deviations from the mean; originally attributed to human error) made an almost full recovery over the course of the year. One cell went from an original PCE of 0.85–4.1%. Again, this was likely due to poor initial penetration of the electrolyte solution, and so after enough time, it was able to form a much better interfacial contact.

The cells were also tested under white light-emitting diode (LED) light (1000 lux/250.8 μW cm^–2^). The value of 250.8 μW cm^–2^ was calculated by measuring the cell at 1000 lux, then using a conversion factor attained through spectral integration as detailed by Michaels in his PhD thesis.^23^ The spectrum of the LED (used for the spectral integration process) is shown in Figure S2. LED results for each cell tested are shown in Table S3. The chosen RZ cell achieved 18.9% PCE, while the SZ cell achieved 21.5%. Their solar simulator efficiencies were 4.7 and 5.0%, respectively (both the LED and solar simulator values were recorded after 12 months of dark storage). The fill factor of the RZ cell increased from 0.65 to 0.75, while the SZ cell increased from 0.59 to 0.78. The increase in fill factor is expected for lower light due to a lower concentration of charge carriers and thus easier charge extraction at the interfaces. Overall, the performance of these cells under low light was very good and, with further optimization, may be suitable for indoor applications.

Due to the fast drying method, we considered the possibility that the RZ cells may have contained trapped solvent, which could have contributed to the observed higher performance due to remaining semiliquid. Tanaka et al. used infrared (IR) spectroscopy to show that their solutions were fully dry through the disappearance of the C≡N peak at 2250 cm^–1^, which is characteristic of MeCN.^10^ We similarly took an IR spectrum of an electrode directly prior to cell assembly (i.e., an electrode with electrolyte deposited onto TiO_2_ in air) to ensure it was fully dry (see Figure S7). We found a complete disappearance of the 2250 cm^–1^ peak, indicating that the electrolyte was fully dried when the RZ cells were assembled.

To substantiate our claim that these are indeed solid-state cells, it is important to first consider the way RZ cells differ from gelation-based cells, which have been developed previously, as both involve the insertion of a liquid electrolyte with a subsequent increase in viscosity. In gelation methods, the electrolyte for RZ cells is usually injected into a hole in the counter electrode,^24−26^ but for RZ cells, it was instead applied directly before assembly to enable the rapid fabrication required for a scalable process. Additionally, we determined that while our electrolyte indeed had an increase in viscosity, it was to an extent to which it can be considered as solid when defined as nonflowing over a reasonable timescale (in this case, 24 h). To demonstrate this, 1 mL of the same solution used in RZ and SZ cells was placed into a sample vial and allowed to dry overnight. IR spectroscopy showed that the electrodes were completely free of MeCN and fully dried. After the drying period, the vial was upturned and left for a day. No flow was observed at this time (Figure S8), so it was concluded that the RZ cells could reasonably be described as solid-state cells. Importantly, however, the good contact obtained with the counter electrode when completing the cells suggests that, while solid, the polyiodide HTM remains somewhat malleable.

Electrochemical impedance spectroscopy [EIS] was performed on each cell type to investigate the interfacial resistances. The values of *R*_rec_ at *V*_OC_ (derived from EIS—see Figure S3) ranged from 14 Ω cm^2^ in the RZ (630 mV) to 47 Ω cm^2^ in the SZ cell (760 mV) and 339 Ω cm^2^ in the Liq cell (830 mV) (Figure S5). Therefore, the Liq cells had the highest *R*_rec_, followed by SZ, then RZ, and these differences would be even more apparent if compared at the same potential. A higher value of *R*_rec_ corresponds to a lower rate of recombination, meaning the RZ cells had the highest rate of recombination out of the three cell types. This observation may be attributed to a combination of lower charge mobility in the higher-order polyiodides and better interfacial contact with the mesoporous electrode.

**Figure 5:**
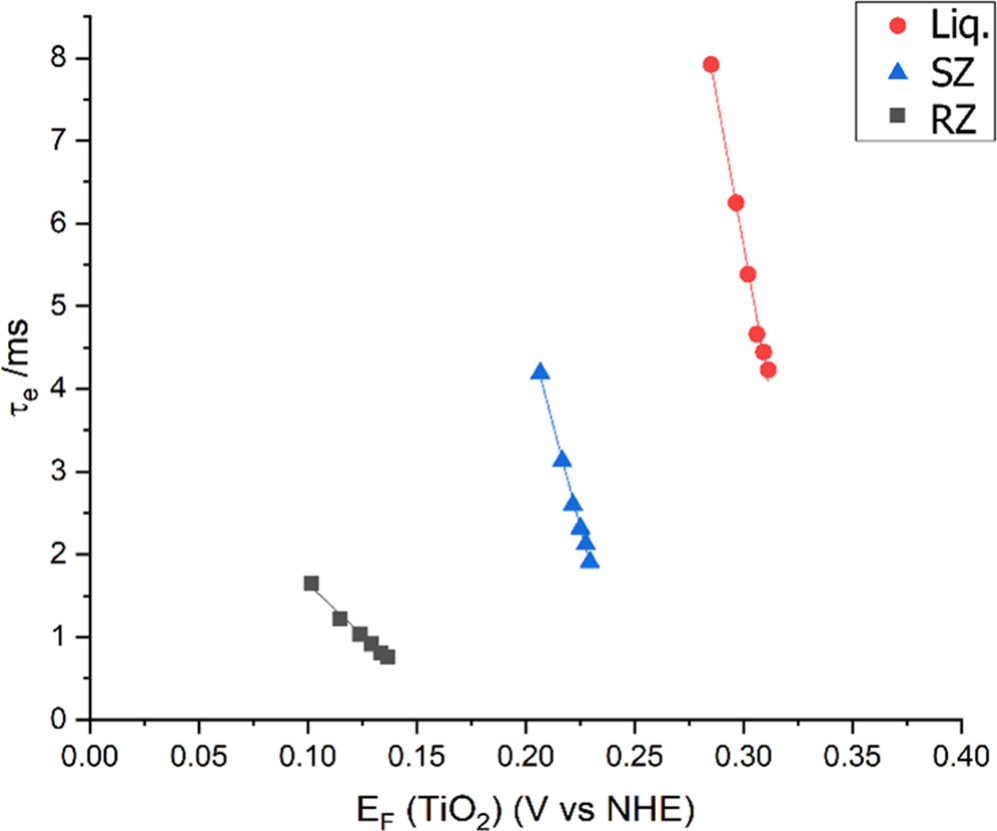
Electron lifetime measurements for representative Liq, SZ, and RZ cells. *E*_f_ (TiO_2_) = *V* – *E*_red_ (electrolyte), where *E*_red_ (electrolyte) is adjusted using the Nernst equation according to the electrolyte formula used.

The recombination kinetics within the cells was further investigated by transient photovoltage analysis (Figure 5). The recombination lifetimes τ_e_ (calculated using eq 1) showed a clear trend from RZ < SZ < Liq. A lower τ_e_ indicates a higher rate of recombination, so the RZ cells were confirmed to have the highest rate of recombination, in agreement with the findings from EIS data and the trend in *V*_OC_ values for the cell types.
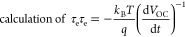
1

**Figure 6:**
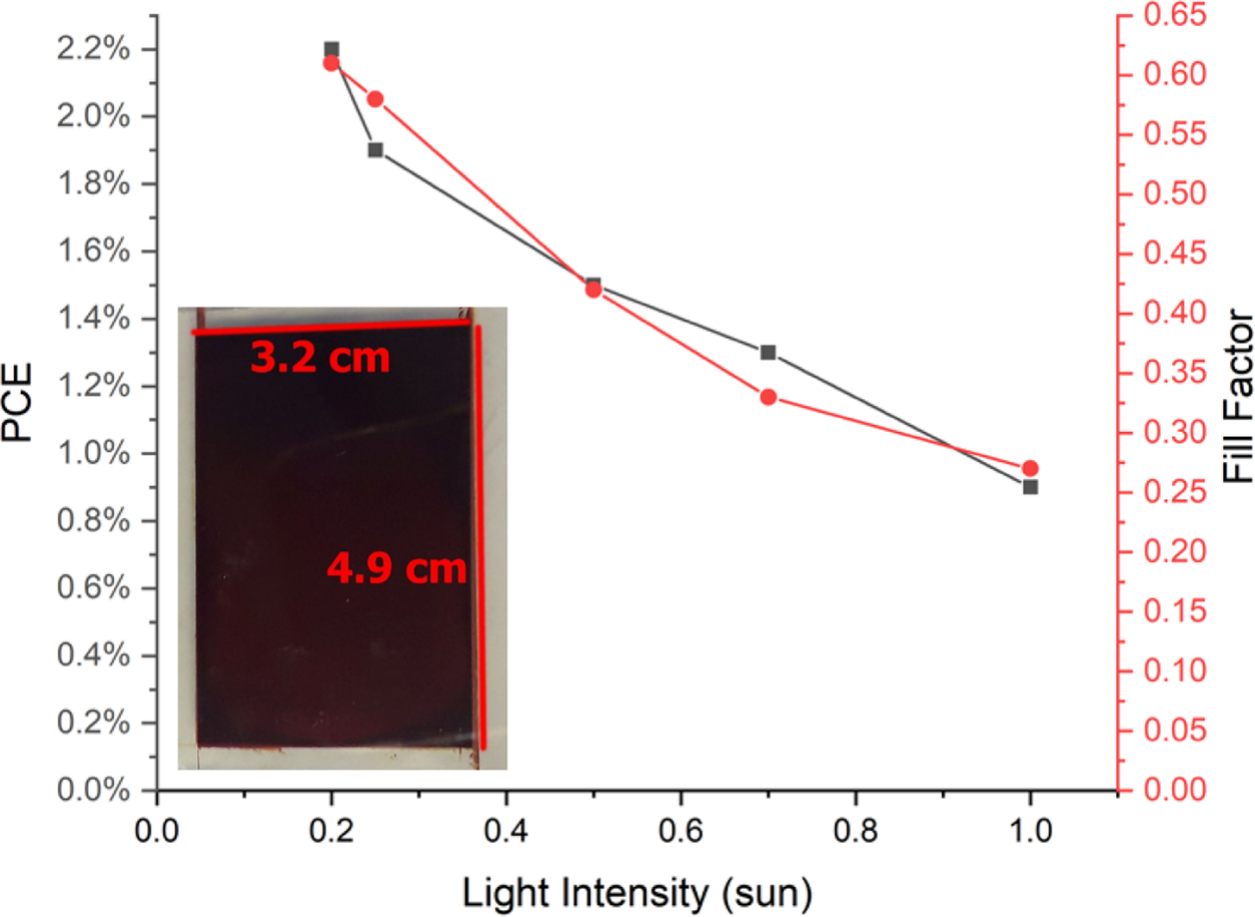
Inverse relationship of fill factor and PCE to light intensity in large-scale cells using a 2.5 cm × 2.5 cm square mask. Inset is a large-area cell with an active area of 3.2 cm × 4.9 cm. Electrolyte was dropped directly onto the active area and allowed to dry for 1 h.

To demonstrate the ease of fabrication and scale-up of these devices, large-area cells of dimensions 3.2 by 4.9 cm, with a total area of 15.68 cm^2^, were fabricated. The fill factor of these cells was decidedly lower than the small-scale cells. This was expected due to a longer pathway for charge collection, hence a much higher concentration of charge carriers. As such, we tested the cells under lower-light conditions and found an inverse relationship between the fill factor (and thus PCE, since *V*_OC_ and *J*_SC_ were mostly unchanging) and the light intensity, which supported the evidence found for the LED measurements. This effect is illustrated in Figure 6, with *J*–*V* curves in Figure S9, and led to PCE over 2% at 0.2 suns. Since these cells are designed for use in low light and indoor conditions (<0.1 sun), the fill-factor loss would not be significantly detrimental, even in such large-area cells. It may also be possible to include silver lines in the future to reduce current losses for a larger area of the cells.

## Conclusions

In conclusion, a rapid and easy drying method was used to produce polyiodide-based zombie DSSCs with an average efficiency of 4.1 ± 0.97% and champion 5.0%, which surpassed the equivalent SZ cells with an average of 2.8 ± 1.2% and champion of 4.5%. They also show promising initial PCE results under indoor conditions using LED lighting, reaching 18.9%. These cells have a higher potential for practical manufacturing due to direct deposition of the HTM and counter electrode enabling rapid fabrication. While these cells outperformed traditional zombie cells with polyiodide electrolytes, the performance may even rival that of liquid cells if the method is refined, and the issue of high rates of recombination can be addressed to improve their performance. Such methods could include further exploration of sterically blocking dyes such as the work carried out by Feldt et al. in 2010,^27^ coating TiO_2_ with a thin inorganic film according to Yao et al. in 2016 with NiO/Eu^3+^, Tb^3+^,^28^ or through cosenitization with a complementary dye such as was done by Luo et al. in 2018.^29^ Other potential expansions on this work could include the use of copper-based electrolytes such as those used by Freitag et al. (CIT) in their original zombie cells, or the use of inorganic dyes such as ruthenium-based complexes to potentially improve their performance. Additionally, alternative electrolytes which are more transparent when dried could also reduce parasitic absorption of incoming light, so they are worthy of investigation. RZ cells were shown to be highly stable compared to both SZ and Liq equivalents. Due to this fact and their very facile and robust fabrication, we believe such further investigation into this method is well warranted.

## Supporting Information Available

Working electrode of an RZ cell, with scotch tape mask applied; spectrum of white LED (5000 K) used for performing indoor light measurements on cells; example of EIS data with fit, showing where the *R*_rec_ value was derived from; equivalent circuit used as a model for EIS; applied voltage during EIS measurements vs extracted value of *R*_rec_ for a representative cell of each type; IPCE and the corresponding integrated photocurrent density of a representative Liq cell; IR spectrum of *I*^–^/*I*_3_^–^ electrolyte, dried for 30 min, on a TiO_2_ working electrode base; sample vials containing undried zombie electrolyte solution, zombie electrolyte solution dried for 24h and dried zombie electrolyte solution which has been upturned for 24h; *J*–*V* curve of large-area cell (Figures S1–S9); average performance metrics of each cell type, with standard deviations; new performance metrics of each cell type after 12 months dark storage, with standard deviations; and performance metrics under LED for representative zombie cells after 12 months dark storage (Tables S1–S3) (PDF)

## Abbreviations

DSSC: dye-sensitized solar cell
PCE: power conversion efficiency
HTM: hole transport material
EIS: electrochemical impedance spectroscopy
SZ: slow-zombie
RZ: rapid-zombie
