# Supplementary material for: Rapid and Facile Fabrication of Polyiodide Solid-State Dye-Sensitized Solar Cells Using Ambient Air Drying
Source: ACS Appl Mater Interfaces. 2022 Sep 16;14(38):43456–62. doi: 10.1021/acsami.2c14299 (PMC9523705; doi:10.1021/acsami.2c14299)
Supplement: Supplementary file 1 — am2c14299_si_001.pdf [file am2c14299_si_001.pdf]

Supporting Information

Rapid and Facile Fabrication of Polyiodide Solid-State Dye-Sensitized Solar Cells Using Ambient Air Drying

Matthew Sutton,[a] Bingyu Lei,[a] Hannes Michaels,[bc] Marina Freitag[bc] and Neil Robertson*[a]

[a] School of Chemistry, The University of Edinburgh, David Brewster Road, Edinburgh, EH9 3FJ, UK.

[b] Department of Chemistry, Ångström Laboratory, Uppsala University, P.O. Box 523, SE-75120 Uppsala, Sweden

[c] School of Natural and Environmental Science, Bedson Building, Newcastle University, NE1 7RU, Newcastle upon Tyne, UK

Corresponding Author

* Neil Robertson - Neil.Robertson@ed.ac.uk

Additional Experimental Information

Fluorine-doped tin oxide (FTO) conductive glass (Merck, 7 Ω sq^{-1}) was cut into 3 cm x 4.2 cm rectangles (two for every 4 cells). Half of the FTO/glass was sonicated in an Ultrawave U300H in:

1) 2% Hellmanex detergent (Hellma) in ultrapure water –30 minutes
2) Ultrapure water –15 minutes
3) Ethanol –15 minutes

The FTO/glass was UV treated for 20 minutes, followed by formation of a compact TiO_2 layer by chemical bath deposition in 40 mM $TiCl_4$ in ultrapure water at 70 °C for 30 minutes. The FTO/glass was sintered at 500 °C for 30 minutes (2 °C per minute ramp). Commercial pastes Ti-Nanoxide T/SP and Ti-Nanoxide R/SP (Solaronix) were screen-printed in a 0.2827 cm^2 (6 mm diameter) circular area, followed by sintering at 500 °C for 15 minutes (1 °C per minute ramp). The electrodes were again treated in 40 mM $TiCl_4$ in ultrapure water at 70 °C for 30 minutes, followed by sintering at 500 °C for 30 minutes (2 °C per minute ramp). The electrodes were cut into quarters, giving four 1.5 cm x 2.1 cm rectangular working electrodes. While still warm (~80 °C), the electrodes were introduced into dye baths and left for at least 12 hours in dark storage. The dye baths were comprised of 0.1 mM LEG4 dye and 0.4 mM chenodeoxycholic acid in 1:1 (v/v) MeCN/tert-butanol.

The remaining FTO/glass had two 1 mm diameter holes drilled in each electrode. One in the corner 0.5 cm from each respective edge, and another beside it 0.5 cm from the opposite edge. This drilling step was ignored for RZ cells. Platisol T/SP (Solaronix) was doctor-bladed onto the FTO (covered full area of the electrode), followed by annealing at 450 °C for 15 minutes (2 °C per minute ramp).

For liq. and SZ cells, the electrodes were clamped and sealed together with ThreeBond UV-curing glue (product code TB3027G). The electrolyte was then injected through the open holes in the

counter-electrode. The electrolyte used in the Liq cells was as follows: I_2 (0.05 M), LiI (0.1 M), 1,2-dimethyl-3-propylimidazolium iodide [DMPII] (0.6 M), anhydrous acetonitrile [ACN] (4.63 mL) and 4-tert-butylpyridine [TBP] (0.5 M, 0.37 mL). The SZ and RZ cell electrolytes were based on this formula, with both having double the I_2 concentration (0.1 M I_2) of the liq. recipe. For the SZ cells the electrode holes were sealed after 2 days in dark storage, and in the case of the Liq. cells they were sealed immediately. The dyed working electrodes of the RZ cells were masked using scotch tape as shown in Fig. S1, with a 7mm diameter circle cut from the centre for the active area. 2 µL of the electrolyte solution was then pipetted evenly across the surface of the exposed active area and allowed to dry for thirty minutes in air. After drying, the tape was removed and the cell was sealed with Araldite Rapid 5 Min 2 component epoxy resin. This was used in place of the UV-curing glue since there was no need to fully seal the cell as it was already fully dry, and the epoxy resin was a cheaper and easier solution for holding the electrodes together.

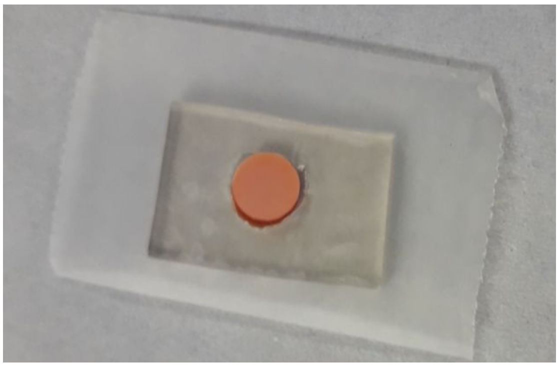

Figure S1: Working electrode of a RZ cell, with scotch tape mask applied. Electrolyte would be added onto the active area and allowed to dry.

Large-area cells were fabricated by cutting FTO/glass into sections of 4.4 x 6.5 cm. Ti-Nanoxide T/SP and Ti-Nanoxide R/SP (Solaronix) were doctor-bladed onto a 3.2 cm x 4.9 cm area. The active area was masked with strips of scotch tape on the edges, and 200 µL of electrolyte was spread evenly on the active area and was allowed to dry for 1 hour. After drying, the tape was removed and the cell was sealed using Araldite Rapid 5 Min 2 component epoxy resin.

Electrochemical Impedance Spectroscopy

EIS was measured using Autolab PGStat 30 potentiostat with Autolab Frequency Response Analyser v4.9.007. Measurements were taken using a white LED and 0.5 mm diameter mask to gather a spread of 4 sets of data from around $V_{oc}/2$ to just above V_{oc}. Points were recorded from 100 kHz to 0.01 Hz, with 50 points in alogarithmic distribution, and an amplitude (rms) of 0.01 V. Data was fitted and R_{rec} was extracted using Scribner Associates Inc. ZView2 software.

J-V Curves

J-V curves were measured using Autolab PGStat 30 potentiostat with Autolab General Purpose Electrochemical System v4.9.007. Measurements were taken from 0.02 V to -1 V with a step

potential of 0.00244 V and a scan rate of 0.05 Vs^{-1}. For all cells with a 6mm diameter active area, a 3 mm diameter circular mask was used. Large-area cells were tested with both a 2.5 x 2.5 cm square mask and a 3 mm diameter circular mask. J-V curves were also recorded for 12 of the final cells using a Voss electronic GmbH HelloSim-CL60 solar simulator and IV Measure v2.3 (©Hannes Michaels) in Newcastle University. Measurements were recorded from -0.1 V to 0.9 V with a step potential of 5 mV and a scan rate of 100 mV s^{-1}. A 4 x 4 mm square mask was used for these cells.

Transient photovoltage measurements

Electron recombination lifetimes were investigated using a 1 W white LED with the *Toolbox* from Dyenamo (Stockholm). Kinetics in the solar cell were probed by applying square-wave modulations to the light intensity. The solar cell voltage response was tracked by a digital acquisition board and fitted with first-order kinetic models. Measurements were recorded at LED currents 200, 300, 400, 500, 600 and 700 mA. 700 mA approximately corresponded with 100 mW cm^{-2} light intensity. 10,000 samples/s were recorded with 20 Hz repetition frequency.

Data were collected as V_{OC} vs τ_e (s). V_{OC} was adjusted to be E_f (TiO_2) = V – E_{red} (electrolyte), where E_{red} (electrolyte) is adjusted using the Nernst equation (Equation 1) according to the electrolyte formula used (Equations 2 and 3 for Liq. and zombie, respectively).

$$E = -E^{\emptyset} + \frac{RT}{2F} ln\frac{[I_3{}^-]}{[I^-]^3} \qquad (1)$$

$E^{\emptyset}$ = -0.53 for I^-/I_3^-

$$E_{liq} = -0.53 + \frac{8.314(298)}{2(96485)} ln\frac{0.05}{0.65^3} = -0.552\ V \qquad (2)$$

$$E_{zom} = -0.53 + \frac{8.314(298)}{2(96485)} ln\frac{0.05}{0.55^3} = -0.545\ V \qquad (3)$$

Incident-photon-to-current-conversion efficiency (IPCE)

For quantum efficiency spectra, the cells were illuminated with light from a Newport 69902 Xenon lamp monochromated by an Oriel Cornerstone 130 1/8m monochromator. The photocurrents were recorded using an Ivium Compactstat. The setup was calibrated with a certified silicon reference cell from Fraunhofer. The photocurrents were integrated based on the spectral distribution of sunlight (AM1.5G).[Reference Air Mass 1.5 Spectra. https://www.nrel.gov/grid/solar-resource/ spectra-am1.5.html. Accessed: 2018-03-28.]

Infrared Spectra

Data was collected on a Perkin Elmer UATR Two with Perkin Elmer Spectrum software. Data was collected from 4000 cm^{-1} to 1500 cm^{-1} at 0.5 cm^{-1} resolution with 4 scans

Average Performance Metrics of Cells and Dark Storage Stability

Cell Type	J_{SC} (mA cm^{-2})	V_{OC} (V)	Fill Factor	PCE (%)
Liq.	11.9 ±0.463	0.765 ±0.0164	0.739 ±0.0158	6.72 ±0.141
SZ	7.43 ±2.04	0.703 ±0.0165	0.510 ±0.125	2.79 ±1.22
RZ	9.84 ±1.55	0.603 ±0.0333	0.678 ±0.117	4.11 ±0.970

Table S1: Average performance metrics of each cell type, with standard deviations

Cell Type	J_{SC} (mA cm^{-2})	V_{OC} (V)	Fill Factor	PCE (%)
Liq.	4.78 ±3.70	0.684 ±0.0217	0.456 ±0.0773	1.37 ±0.969
SZ	10.6 ±2.82	0.620 ±0.0209	0.401 ±0.115	2.78 ±1.31
RZ	10.5 ±1.07	0.669 ±0.0245	0.663 ±0.0344	4.64 ±0.423

Table S2: New performance metrics of each cell type after 12 months dark storage, with standard deviations

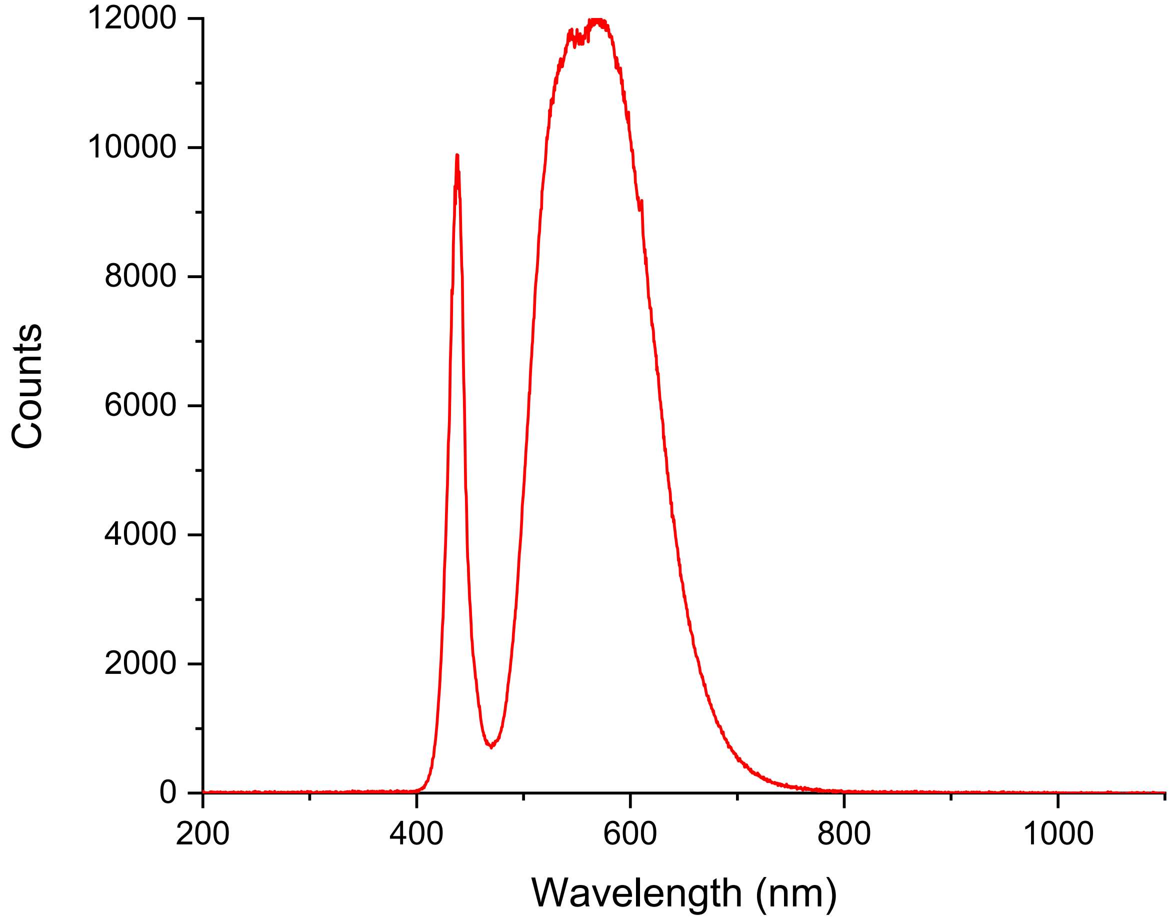

Indoor Light Measurements

Figure S2: Spectrum of white LED (5000K) used for performing indoor light measurements on cells

Cell Type	J_{SC} (µA cm^{-2})	V_{OC} (V)	Fill Factor	PCE (%)
SZ	125.20	0.55	0.78	21.5%
RZ	113.16	0.56	0.75	18.9%

Table S3: Performance metrics under LED for representative zombie cells after 12 months dark storage. Measured under 1000 lux (250.8 µW/cm^{-2}) light intensity

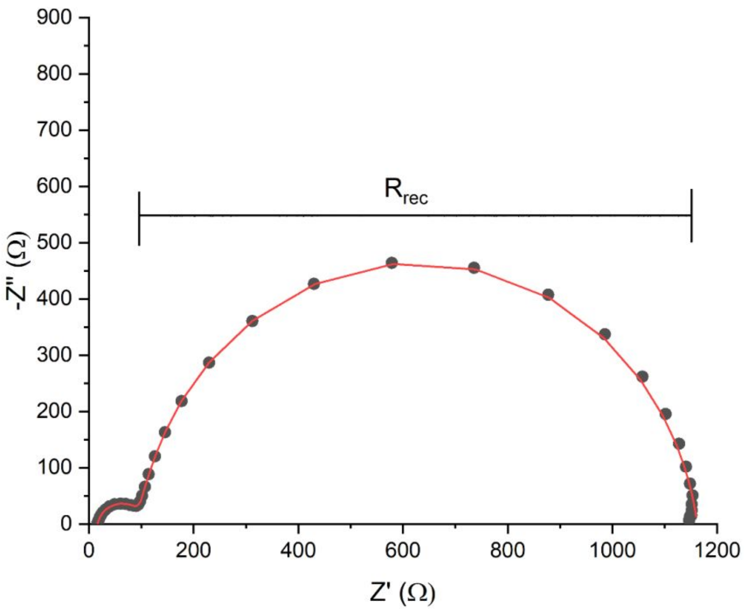

Electrochemical Impedance Spectroscopy

Figure S3: Example of EIS data with fit, showing where the R_{rec} value was derived from. The equivalent circuit used to model this is shown in Fig. S3. R_{rec} values were plotted against applied voltage (Fig. S4)

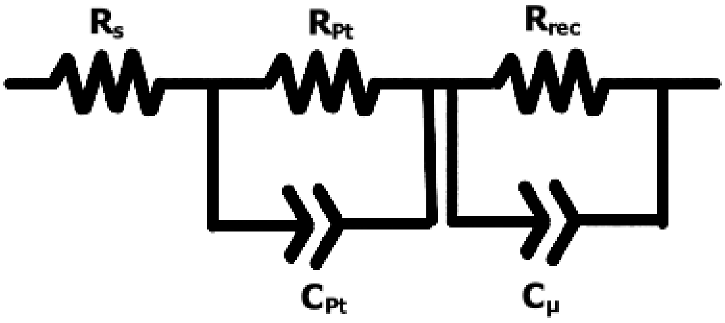

Figure S4: Equivalent circuit used as model for EIS.

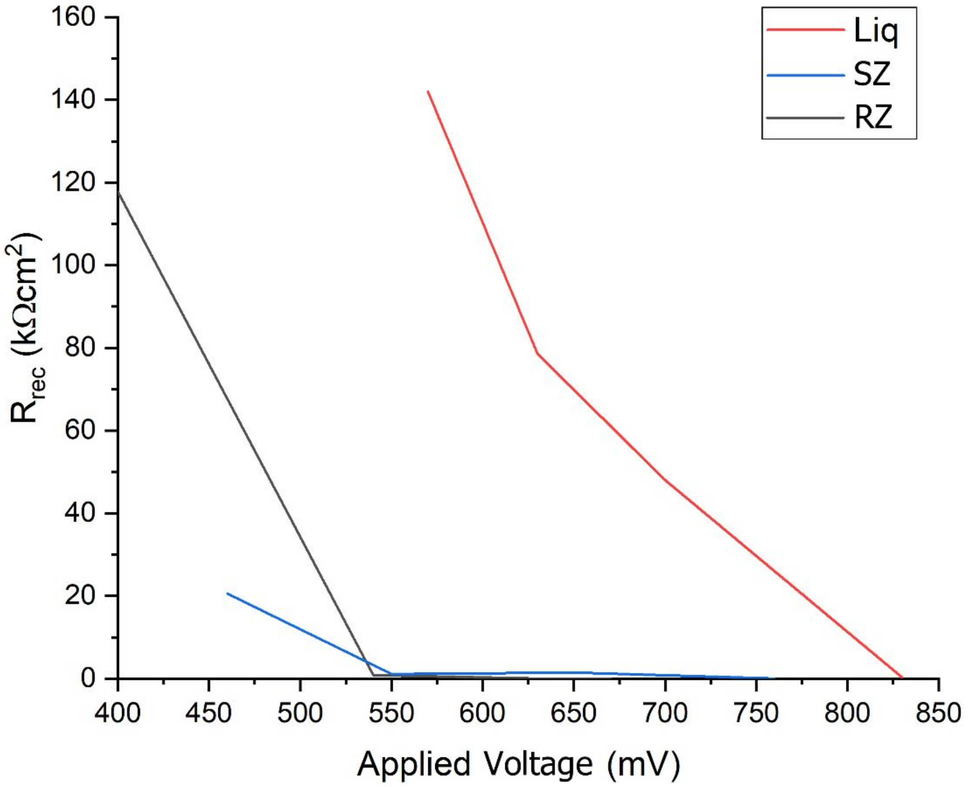

Figure S5: Applied voltage during EIS measurements versus extracted value of R_{rec} for a representative cell of each type

Incident-Photon to Electron Conversion Efficiency (IPCE)

IPCE measurement was used to verify the J_{SC} values acquired from J-V curves.

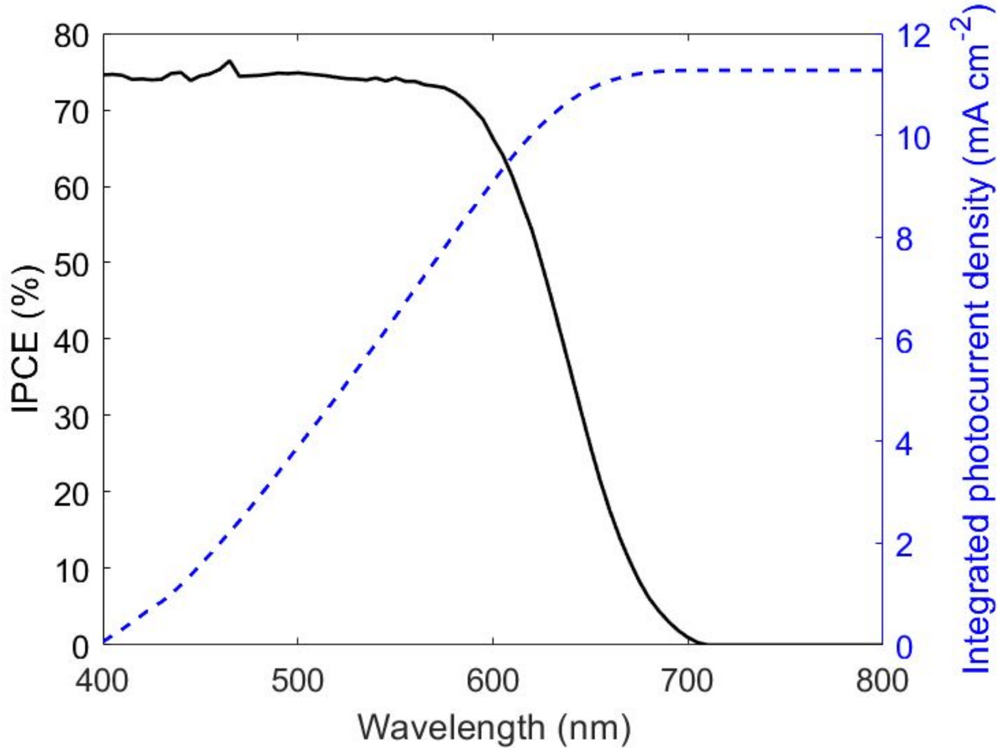

Figure S6: IPCE and the corresponding integrated photocurrent density of a representative Liq. Cell

The value of 11.2 mA/cm^2 acquired from IPCE agreed well with the J-V value of 11.5 mA/cm^2, thus verifying the J-V findings.

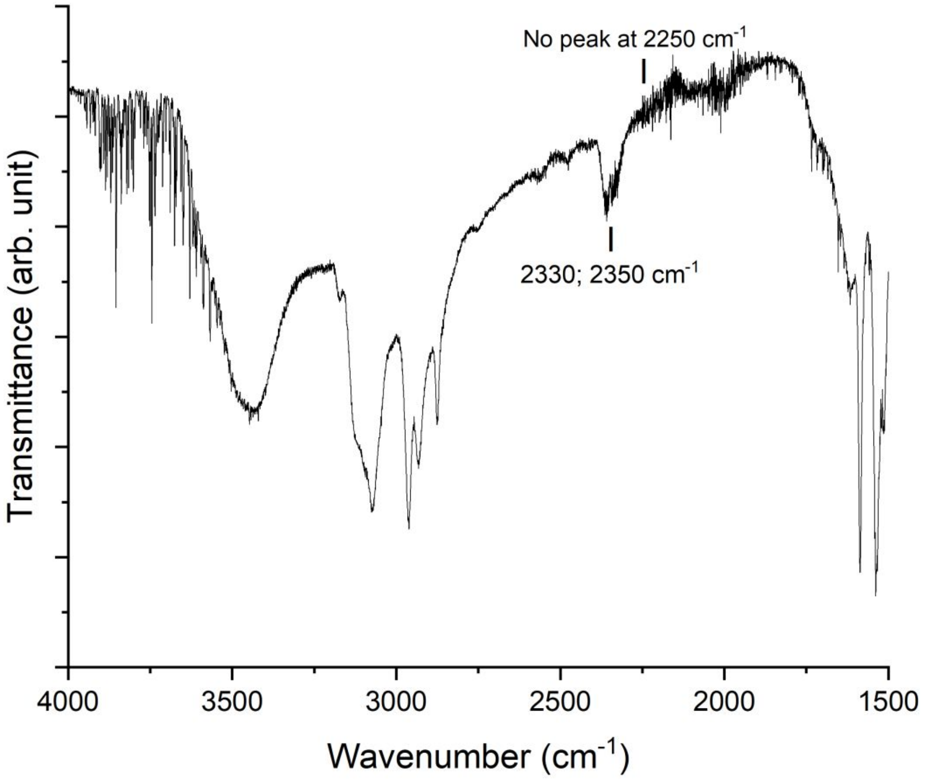

Figure S7: IR Spectrum of I^-/I_3^- electrolyte, dried for 30 mins, on a TiO_2 working electrode base. The characteristic MeCN peak at 2250 cm^{-1} is not present, indicating that the material has been fully dried. Additional peak assignments can be found in previous work - Tanaka, E.; Robertson, N., Polyiodide solid-state dye-sensitized solar cell produced from a standard liquid I^-/I_3^- electrolyte. *Journal of Materials Chemistry A* **2020**, *8* (38), 19991-19999

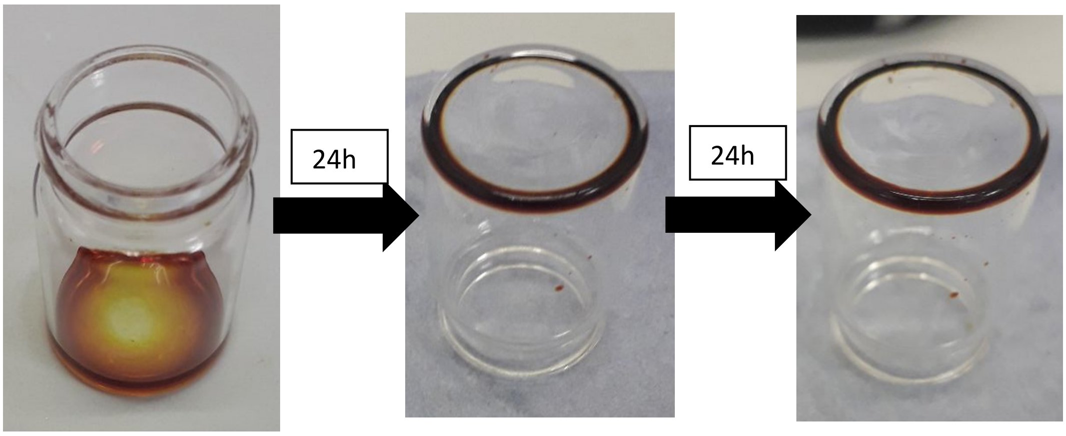

Figure S8: Sample vial containing undried zombie electrolyte solution (left), electrolyte solution dried for 24h (middle) and dried electrolyte solution after being upturned for 24 h (right)

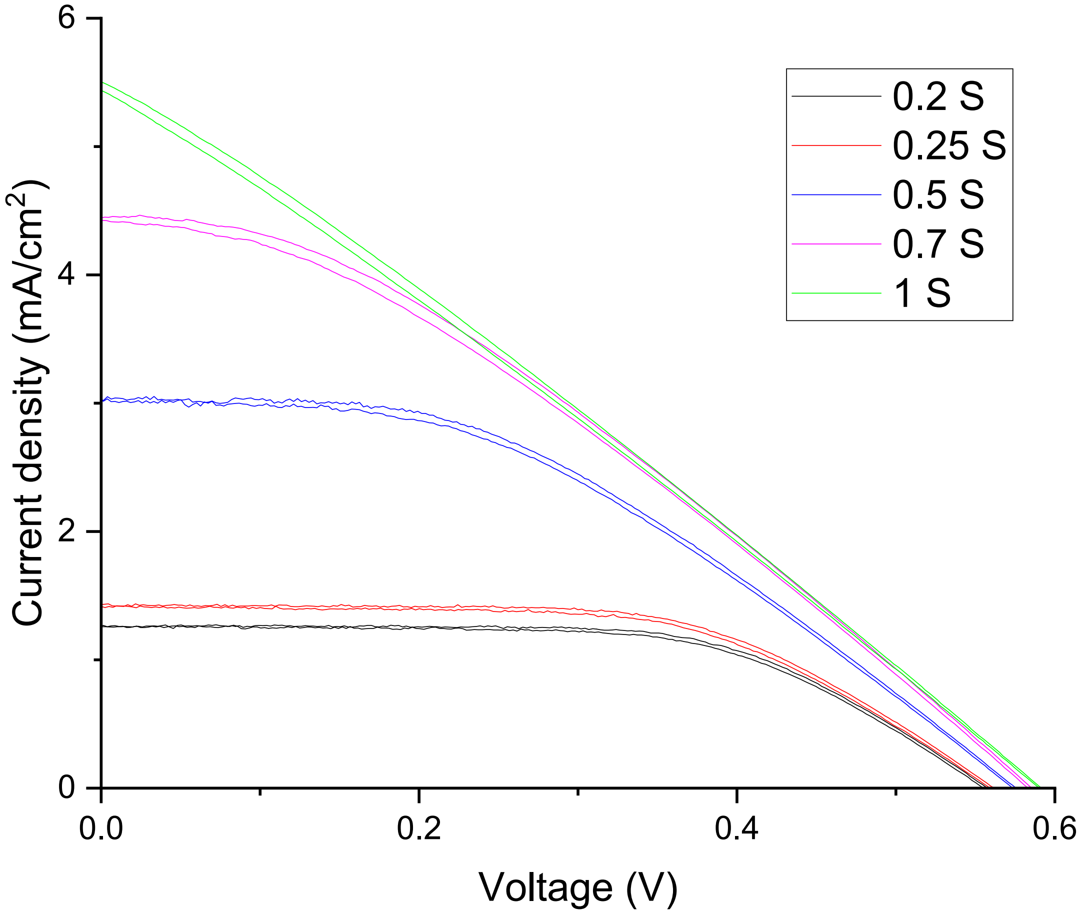

Figure S9: J-V curve of large area cell recorded between 20 (0.2 S) and 100 mW/cm^2 (1 S) using a 2.5 cm side square mask (6.25 cm^2) between 0 and 0.6 V
